# Means of Counteracting the Action of Caustics

**Published:** 1846-06

**Authors:** 


*Means of counteracting the action of caustics.*—When you use a caustic, it is always prudent to have some counter-agent at hand to stop its action if its reaches a sound part. If you use nitric acid, you should have at hand a solution of the bicarbonate of potash; caustic potash may be neutralized by vinegar, or by a solution of the diacetate of lead. If you are afraid of nitrate of silver burning the neighboring parts, its action may be neutralized by common olive oil. A solution of bicarbonate of potash will decompose chloride of zinc, and so on with other caustics.—*Sir B. Brodie's Lectures on Pathology and Surgery.*

[The carbonate will answer as well as the bicarbonate of potash, and a strong solution of common salt will effectually decompose and neutralize the caustic properties of nitrate of silver.—Ed. Gaz.]

